# Cell Fusion-Mediated Tissue Regeneration as an Inducer of Polyploidy and Aneuploidy

**DOI:** 10.3390/ijms21051811

**Published:** 2020-03-06

**Authors:** Jessica Dörnen, Mareike Sieler, Julian Weiler, Silvia Keil, Thomas Dittmar

**Affiliations:** Institute of Immunology, Center for Biomedical Education and Research (ZBAF), Witten/Herdecke University, 58448 Witten, Germany; jessica.doernen@uni-wh.de (J.D.); mareike.sieler@uni-wh.de (M.S.); julian.weiler@uni-wh.de (J.W.); silvia.keil@uni-wh.de (S.K.)

**Keywords:** stem cells, tissue regeneration, cell fusion, polyploidy, aneuploidy

## Abstract

The biological phenomenon of cell fusion plays a crucial role in several physiological processes, including wound healing and tissue regeneration. Here, it is assumed that bone marrow-derived stem cells (BMSCs) could adopt the specific properties of a different organ by cell fusion, thereby restoring organ function. Cell fusion first results in the production of bi- or multinucleated hybrid cells, which either remain as heterokaryons or undergo ploidy reduction/heterokaryon-to-synkaryon transition (HST), thereby giving rise to mononucleated daughter cells. This process is characterized by a merging of the chromosomes from the previously discrete nuclei and their subsequent random segregation into daughter cells. Due to extra centrosomes concomitant with multipolar spindles, the ploidy reduction/HST could also be associated with chromosome missegregation and, hence, induction of aneuploidy, genomic instability, and even putative chromothripsis. However, while the majority of such hybrids die or become senescent, aneuploidy and genomic instability appear to be tolerated in hepatocytes, possibly for stress-related adaption processes. Likewise, cell fusion-induced aneuploidy and genomic instability could also lead to a malignant conversion of hybrid cells. This can occur during tissue regeneration mediated by BMSC fusion in chronically inflamed tissue, which is a cell fusion-friendly environment, but is also enriched for mutagenic reactive oxygen and nitrogen species.

## 1. Introduction

Approximately 20 years ago, the first papers were published demonstrating that bone marrow-derived stem cells (BMSCs), such as mesenchymal stem/stromal cells (MSCs) and hematopoietic stem cells (HSCs), possess a certain degree of plasticity/pluripotency and could be functionally differentiated into hepatocytes [1,2,3,4,5,6,7], neurons [6,8,9,10,11,12], cardiomyocytes [6,13,14], skeletal muscle [15,16,17], intestinal cells [18,19,20] or insulin-producing cells [21]. Even though it is currently known that BMSCs are not pluripotent, as they were originally thought to be, these findings raised great expectations at that time regarding their use in future tissue regeneration strategies [22,23]. Data showed that BMSCs could transdifferentiate into functional organ cells in accordance with the blueprint provided by the target tissue [24,25], suggesting that BMSCs could be simply administered to the desired tissue, and then regeneration would run in a self-autonomous way. More thorough analyses finally revealed that transdifferentiation of BMSCs (and other stem cells) could be either induced by soluble factors secreted by cells [2,26,27] or could be due to the biological phenomenon of cell fusion [4,5,6,7,8,10,12,18,20,28,29,30,31,32,33]. In particular, the finding that stem cells could adopt the properties of foreign tissue cells by merging with them was confusing. This referred not only to the process of cell fusion itself, which to date is still not well understood but also to the fate of the hybrid cells that were originated. It is well known that bi- or multinucleated hybrid cells (so-called heterokaryons), such as osteoclasts, myofibers, and syncytiotrophoblasts, originate first from such cell fusion events, and then remain stable as heterokaryons in the body [34,35]. In fact, bi- and multinucleated heterokaryons were found in BMSCs that regenerated Purkinje neurons in the brain [6,8,10], and they also found in regenerated myocytes [10], cardiomyocytes [6], and hepatocytes [5,6,7]. However, it is also well known that bi- and multinucleated hybrid cells can undergo ploidy reduction/ heterokaryon-to-synkaryon transition (HST), thereby giving rise to mono- and binucleated cells [36,37,38,39,40,41,42]. Indeed, BMSC-derived mononuclear cells were found in regenerated tissue, which were positive for both donor and recipient markers, indicating that such cells have truly originated from cell fusion [18,29,43,44]. However, the mechanism of ploidy reduction/HST is complex and still not well understood. Ploidy reduction/HST of hybrid cells could either result in daughter cells with a normal diploid karyotype [37,42] or in aneuploid daughter cells that are genomically unstable [7,36,39,45,46,47,48]. Likewise, the fate of (cell fusion-derived) aneuploid and genomically unstable cells is not yet clear. Induction of aneuploidy and genomic instability is commonly associated with cell death or senescence (for review see [47]), but data from BMSC-derived hepatocytes revealed that aneuploidy and genomic instability is tolerated in this cell type and might even be beneficial for stress-related adaptation and regeneration processes [36,39,40,49,50,51]. In contrast, a few studies have been published indicating that cell fusion might also result in the origin of neoplastic cells due to the induction of aneuploidy and genomic instability [52,53,54].

In the present review, we will summarize and discuss the role of cell fusion as an inducer of polyploidy and aneuploidy and the fate of such cell fusion-derived cells.

## 2. How Do Cells Fuse with Each Other?

Although different physiological processes, such as fertilization, placentation, myogenesis, osteoclastogenesis, and tissue regeneration, depend on cell fusion, the mechanism by which two (or more) cells hybridize is still not well understood [34,35,55,56,57]. On the one hand, cell fusion is a tightly regulated process that can be subdivided into five steps: (i) priming, (ii) chemotaxis, (iii) adhesion, (iv) fusion, and (v) postfusion [58]. Cells are not fusogenic per se, so they have to adopt a pro-fusogenic state first in order to fuse with other cells (“priming”). Subsequently, they have to get in close contact with each other (“chemotaxis” and “adhesion”) before they can merge plasma membranes (“fusion”). Finally, they have to return to a non-fusogenic state after the fusion process (“post-fusion”). Several proteins, such as chemokines, cytokines, proteases, adhesion molecules, transmembrane proteins or proteins that are mandatory for actin remodeling, have been identified so far that mediate distinct steps in this cell fusion cascade. However, it remains to be elucidated how cytokines, such as interleukin-4 (IL-4) or receptor activator of NF-κB ligand (RANKL), or proteases, such as matrix metallopeptidase 9 (MMP-9), are exactly involved in the process of cell fusion (for review see [34,35,58]). 

In addition, different cell fusion mechanisms have been developed during evolution. For instance, the fusion of trophoblasts to syncytiotrophoblasts is chiefly regulated by Syncytin-1 and -2, which are transmembrane proteins of retroviral origin [59,60]. Syncytin-1 and -2 are still the best characterized cell fusion mediating proteins in humans, and they might also be involved in human osteoclast fusion [61] and in the fusion of cancer cells with endothelial cells [62,63] or mesenchymal stem cells [64]. In contrast, fusion of myoblasts to multinucleated myofibers depends on remodeling of the actin cytoskeleton and formation of podosome-like structures, which penetrate the target cell, thereby causing the merging of plasma membranes [65,66]. Likewise, several proteins, such as MMP-9, E-Cadherin, Syncytin-1, CD200, dendrocyte expressed seven transmembrane protein (DC-STAMP), osteoclast stimulatory transmembrane protein (OC-STAMP), CD44, and P2X7, have been identified that play a role in macrophage fusion (for review see: [34,35,57]). It is also known that the expression of these proteins is induced by cytokines, such as IL-4 and RANKL [67,68,69,70,71], suggesting that these factors are likely involved in the transition of macrophages from a non-fusogenic to a pro-fusogenic state. Nonetheless, the detailed process of macrophage fusion remains unclear.

Numerous studies further showed that the frequency of cell fusion events was increased upon acute tissue damage or chronic inflammation [7,12,18,32,63,64,72,73,74,75], which is plausible with regard to efficient BMSC-based and cell fusion-mediated tissue regeneration. BMSCs not only have to be converted into a pro-fusogenic state for subsequent hybridization with target cells but also have to be recruited to the site of tissue damage. In this context, it has been shown that the pro-inflammatory cytokine tumor necrosis factor-α (TNF-α) might also be a mediator of cell fusion. Osteoclastogenesis [70,74,75], as well as the fusion of cancer cells with endothelial cells [63,76], mesenchymal stem cells [64], or breast epithelial cells [72,73,77] can be induced by TNF-α. Some data revealed that TNF-α could mediate fusion due to induction of MMP-9 expression [73,75], which plays a role in osteoclastogenesis and giant cell formation [75,78]. Hence, it might be assumed that TNF-α could be involved in cell fusion due to induction of pro-fusogenic proteins and/or in an overall conversion of cells into a pro-fusogenic state. 

In addition to inflammation-induced BMSC-based cell fusion events, two studies revealed that cell fusion events could also occur in the absence of tissue damage and inflammation [18,42]. Using a parabiotic model (a green fluorescent protein (GFP) mouse and a ROSA/β-gal mouse were surgically joined), administration of an anti-inflammatory drug cocktail was found to promote cell fusion-derived GFP/β-Gal positive cells, which were found in approximately 5% of the intestinal crypts of ROSA/β-Gal mice [18]. Likewise, noninflammation-related fusion events were found with a frequency of approximately 0.03 to 0.21% in the murine hematopoietic system [42]. Interestingly, examination of donor and host autosomal reporter genes (*h*CD46, *m*X, CD45.2, GFP, *m*Y, and CD45.1) revealed independent segregation of alleles in more than half of the fusion products, and a loss of parental markers was even observed in some cells [42]. However, despite these genetic changes, neither lineage restriction nor malignant conversion of hematopoietic cells was observed [42]. Whether this indicates that hematopoietic cells might be more tolerant to limited chromosomal sequence gains than other cells [42] remains to be elucidated. 

Both viruses and exomes have also been associated with cell fusion [53,79,80,81]. In vitro and in vivo studies demonstrated that enveloped and non-enveloped viruses could cause cell fusion (so-called fusogenic viruses), thereby giving rise to bi- and multinucleated heterokaryons (a detailed overview of fusogenic viruses is found here [82]). Enveloped viruses, such as HIV, influenza virus or herpesvirus, fuse with the plasma membrane of host cells (for review see [81]) and could cause cell hybridization by acting as bridging particles. For instance, hybridomas derived from plasma cells and myeloma cells were initially generated by using inactivated Sendai virus as a fusogen [83], which has the ability to induce bi- and multinucleated cell formation in vitro and in vivo [84]. Likewise, virus-infected cells could also fuse with other cells due to the expression of viral-derived fusogenic proteins. The nonenveloped fusogenic avian and Nelson Bay reoviruses could induce cell fusion via the expression of so-called fusion-associated small transmembrane (FAST) proteins that are localized in the plasma membrane of infected cells [85]. Binucleated cell formation by fusion was also induced by the human papillomavirus 16 oncogene E5 [86]. 

Exosomes are a type of extracellular vesicle with a diameter of less than 100 nm, and they originate from the invagination of the lipid bilayer of multivesicular bodies in cells (for review see [87,88]). They typically contain tetraspanins (CD9, CD63, CD81, and CD82), heat shock proteins (HSC20, HSP60, HSP70, and HSP909), MHC-I and MHC-II, cell adhesion molecules (P-Selectin, αβ-integrins and annexins), and significant amounts of mRNA, miRNA, and lncRNA (for review see [87,88]). Exosomes play a crucial role in intercellular communication and the regulation of different physiological and pathophysiological conditions, whereby their payload could be delivered to target cells by endocytosis, phagocytosis or membrane fusion [88]. Duelli and colleagues demonstrated that exosomes isolated from virus-infected cells contained viral proteins and exhibited fusogenic properties, suggesting a possible role in cell fusion [53]. Miyado et al. further showed that exosomes might be involved in cell fusion by showing that sperm-egg fusion is mediated by vesicles containing CD9 that are released from the egg and interact with sperm [89]. Because CD9 is a major component of exosomes, the authors concluded that this type of extracellular vesicle was released to mediate fertilization [89]. 

In brief, cell fusion is a tightly regulated but not yet fully understood process. Inflammation can induce cell fusion, which would be necessary for rapid and efficient BMSC-based tissue regeneration. However, cell fusion could also occur spontaneously after being triggered by viruses and/or exosomes. 

## 3. Cell Fusion as an Inducer of Polyploidy, Aneuploidy, and Genomic Instability

Cell fusion first results in the origin of bi- and multinucleated heterokaryons, which can remain stable in this polyploid state, as is seen in osteoclasts, syncytiotrophoblasts, and myofibers [34,35]. However, some heterokaryons can undergo ploidy reduction/HST, thereby giving rise to synkaryons or binucleated daughter cells [36,37,38,39,40,41,42]. It is known that proliferation and resolution of the nuclear membranes are prerequisites for ploidy reduction/HST and subsequent segregation of chromosomes to daughter cells. Frade and colleagues demonstrated that “controlled” and “uncontrolled” ploidy reduction/HST could occur in fusion-derived tetraploid cells [37]. Therefore, daughter cells with a 2n karyotype that had the same genome as the parental cells were derived from “controlled” ploidy reduction/HST, whereas “uncontrolled” ploidy reduction/HST gave rise to daughter cells with a 2n karyotype that is a mix of both fusion partners [37]. Uncontrolled ploidy reduction/HST was also observed in hybrid cells derived from intrahematopoietic cell fusion events [42]. However, in contrast to these findings, other studies revealed that ploidy reduction/HST were associated with chromosome missegregation and induction of aneuploidy [7,36,39,52,54,90]. In addition to karyotypes predicted to result from a fusion between a diploid donor cell and diploid host cells (80,XXYY) or a diploid blood cell and a tetraploid hepatocyte (120,XXXXYY), many aneuploid karyotypes consisting of various combinations of autosomes and sex chromosomes were found in BMSC-derived liver cells [7]. A thorough analysis revealed that a variety of bipolar, tripolar, and double mitoses concomitant with lagging chromosomes occurred in individual BMSC-derived hepatocytes, which resulted in mononucleated, binucleated and aneuploid daughter cells [39]. Therefore, several gains and losses of whole chromosomes were found in BMSC-derived aneuploid hepatocytes [39]. 

Ploidy reduction/HST that was accompanied by induction of aneuploidy was also observed in cell fusion-derived HeLa cell hybrids and rat intestinal epithelial cell hybrids [54]. Tripolar mitosis was observed in a hybrid cell that was derived from HeLa cells with either green- or red-labeled chromosomes [54]. Thereby, time-lapse images showed that green and red labeled chromosomes were randomly segregated, which resulted in three mononuclear daughter cells with a mixed green and red karyotype [54]. Moreover, rat epithelial cell hybrids with a near triploid or tetraploid karyotype became near diploid with repeated passages [54]. Whether this occurred because of additional ploidy reduction/HST or other mechanisms in these hybrids remains unclear. In any case, metaphase spreads as well as γ-H2AX staining revealed an increased frequency of DNA damage, including Robertsonian translocations, in these hybrids [54]. Moreover, animal studies revealed that some hybrids were capable of inducing tumors, indicating that these cells had undergone malignant transformation [54]. An increased frequency of chromosomal aberrations, such as gains, losses and translocations, was also observed in highly aneuploid and tumorigenic hybrids that were derived from virus-mediated cell fusion events of normal fibroblasts with a diploid karyotype [90]. Likewise, highly aneuploid and genomic instable hybrid clones originated from fusion events between IMR90 E6-E7-HRASG12V-CFP (R-CFP) fibroblasts with IMR90 E6-E7-SmallT-hTERT-DsRed (ST-DsRed) fibroblasts [52]. Copy number variation and allele frequency analyses as well as multicolor metaphase spreads revealed a rearranged genome in hybrid cells, including gains, losses and translocation of chromosomes [52]. Briefly, these findings indicate that fusion of non-transformed cells can result in neoplastic hybrids. However, appropriate in vivo data are still missing, and because of that, it remains unclear whether neoplastic cells can truly originate from such fusion events. In fact, this is similar to the possible correlation between cytokinesis defects, induction of polyploidy and cancer (for reviews see: [91,92]). Cytokinesis is a multistep process that can be subdivided into (i) furrow ingression, (ii) furrow constriction, (iii) midbody formation and (iv) abscission [92]. Each step is directed by a specific subset of proteins, and it is known that inhibition or excessive activation of different cytokinesis components can cause cytokinesis defects and the origin of polyploid cells [92]. Even physical obstructions induced by, for instance, asbestos fibers or an invaded cell (entosis), can cause cytokinesis defects and polyploidy [91]. Because there is no difference between cell fusion-induced polyploidy and defective cytokinesis-induced polyploidy, the outcome for the emerging heterokaryons would be similar.

**Figure 1:**
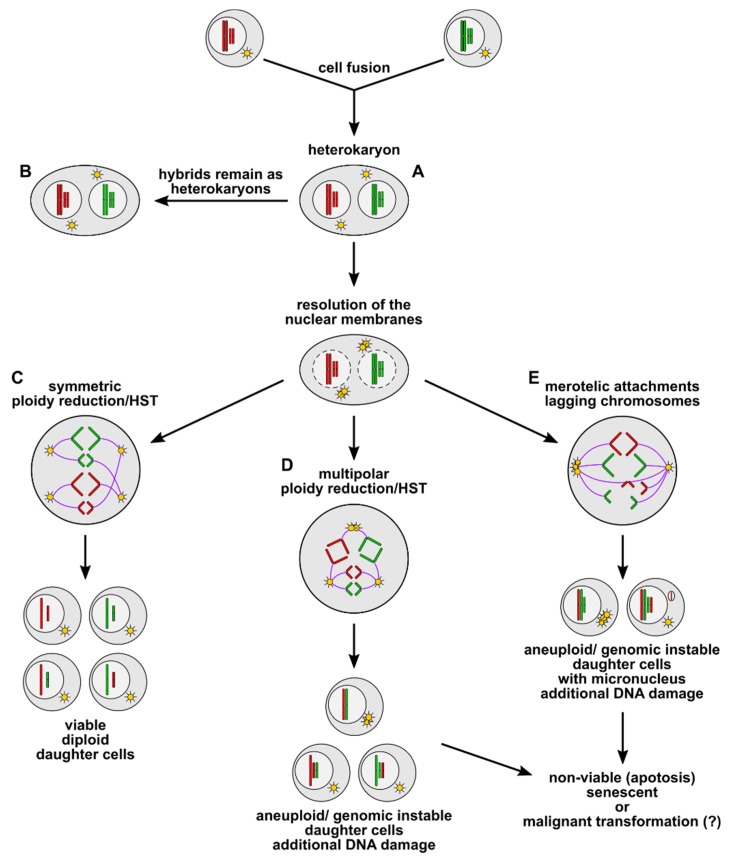
Cell fusion induced polyploidy, aneuploidy, and genomic instability. Cell fusion first results in the origin of bi- or multinucleated heterokaryons (**A**), which could either remain in this state (**B**) or could undergo ploidy reduction/heterokaryon-to-synkaryon transition (HST). Symmetric ploidy reduction/HST (**C**) gives rise to viable diploid daughter cells, whereby a random segregation of parental alleles to daughter cells can be possible. Multipolar ploidy reduction/HST (**D**) results in the origin of aneuploid/genomic unstable daughter cells, whereas merotelic attachments (**E**) during mitosis can cause lagging chromosomes and micronucleus formation in aneuploid/genomically unstable daughter cells. Aneuploidy/genomic instability concomitant with micronucleus formation is associated with further DNA damage, such as chromothripsis and translocations (not shown here). Most of these aneuploid/genetically unstable cells will be nonviable or will become senescent. However, it cannot be ruled out that some hybrid cells will survive and that aneuploidy/genomic instability concomitant with further DNA aberrations could ultimately lead to a malignant transformation of the cells.

It is well known that DNA damage and chromosomal structural aberrations, such as translocations, are closely related to chromosome missegregation during mitosis [93]; such aberrations could be the result of extra centrosomes and merotelic-kinetochore-microtubule attachment errors, which can occur in polyploid cells [94,95,96,97,98]. Extra centrosomes in cells can originate by several mechanisms, including centrosome overduplication, de novo synthesis of centrosomes, mitotic slippage, cytokinesis defects, and cell fusion (for review see [91,92,99]). Merotelic-kinetochore-microtubule attachment errors occur when microtubules emanating from two centrosomes attach to a single kinetochore of one chromosome and result in so-called “lagging chromosomes” [98,100]. Such chromosomes remain at the spindle equator and are not enclosed in the main nucleus; rather, they form separate micronuclei after mitosis [100]. DNA replication in micronuclei is different from replication in the main nucleus. This is attributed to the ruptured micronuclei membrane resulting both in an efflux of nuclear contents, such as polymerases and nucleotides, and in an influx of cytosolic components, including exo- and endonucleases [101,102]. As a consequence, DNA replication is impaired and aberrant in micronuclei, which results in DNA intermediates rather than in intact chromosomes [101,102] Moreover, micronucleic DNA structures are additionally prone to DNA double strand breaks due to the influx of cytosolic nucleases [101,102,103]. Recently, He and colleagues demonstrated that micronuclei-derived DNA intermediates and fragments are missegregated again at the subsequent round of mitosis, thereby triggering the cells’ overall genomic instability [94]. Moreover, micronuclei-derived DNA fragments inside the newly formed daughter cell nucleus could be reassembled through error-prone nonhomologous end joining due to activation of DNA damage repair mechanisms [103,104,105,106]. Hence, it can be assumed that cell fusion-mediated missegregation of chromosomes might be related to chromothripsis (for reviews see: [103,104,105,106]). Chromothripsis is a single catastrophic event in which missegregated chromosomes inside a micronucleus are scattered into ten to a thousand DNA fragments, which are subsequently reassembled in a random order to give rise to derivative chromosomes with extensive rearrangement [103,104,105,106]. Non-integrated DNA fragments could become inevitably lost or could self-ligate into circular DNA structures called double minutes [103,104,105,106]. In summary, these findings indicate that cell fusion could not only be associated with induction of aneuploidy but also with genomic instability and possibly even with a malignant conversion of cells (Figure 1). 

## 4. What Is the Fate of Cell Fusion-Derived Aneuploid and Genomic Instable Hybrid Cells?

In fact, this question is difficult to answer due to contradictory results. On the one hand, aneuploidy has been associated with cancer and genomic instability, and it has even been putatively linked to the neoplastic transformation of cells [45,46,47,48]. Indeed, a few studies have already demonstrated that the fusion of nontumorigenic cells could truly give rise to tumorigenic hybrids [52,53,54]. Hence, the possibility that cancer may have its origin in an initial cell fusion event cannot be ruled out completely. 

Whether a malignant transformation could also occur in BMSC-derived hybrids remains unclear, since the appropriate studies have not yet been performed. However, it cannot be ruled out that neoplastic cells might originate from BMSC-derived hybrids in chronically inflamed tissue. The chronically inflamed microenvironment is characterized by increased levels of leukocyte and phagocyte-derived reactive oxygen and nitrogen species, which form peroxynitrite, a well-known mutagenic agent [107]. Hence, long-term exposure of BMSC-derived hybrid cells to these highly reactive oxygen and nitrogen radicals could result in permanent genomic alterations such as point mutations, deletions, and even rearrangements [108]. The strongest association of chronic inflammation with malignant diseases is in colon carcinogenesis arising in individuals with inflammatory bowel diseases such as chronic ulcerative colitis and Crohn’s disease [108]. In this context, Davies and colleagues demonstrated that significantly increased cell fusion frequencies were detected in the proliferating epithelium of inflamed intestinal tissue [18]. Furthermore, the authors concluded that cell fusion may potentially impact inflammatory disease pathogenesis, including bowel disease and even cancer [18].

In contrast, data from BMSC-derived hepatocytes suggest that aneuploidy and genomic instability are not associated with cellular transformation [36,39,40,49]. Aneuploidy is frequently found in mouse and human hepatocytes [36,39,49], and murine data revealed that the number of aneuploid hepatic cells increased with age [39]. Recently, Matsumoto and colleagues demonstrated that polyploid hepatocytes could undergo ploidy reduction/HST and subsequent re-polyploidization [40], indicating that putative aneuploid hepatocytes were viable and could proliferate. The reason why aneuploidy is obviously tolerated in hepatic cells remains unclear. Conceivably, cell fusion-induced aneuploidy could be a mechanism for stress-induced liver adaptation [50]. Tyrosinemia type I is attributed to a mutation in the fumarylacetoacetate hydrolase gene (*Fah^-/-^*), and it is known that a loss/mutation in the homogentisic acid dioxygenase (Hgd) gene located on chromosome 16 is protective against this disease. Interestingly, nodules of Hgd-null hepatocytes lacking chromosome 16 rapidly emerged in adult *Hgd+/- Fah-/-* mice that were exposed to chronic liver damage [50]. Likewise, transgenic mice that had a polyploidization defect and whose liver cells were mainly diploid were more susceptible to morbidities and death associated with tyrosinemia-induced liver failure than control mice [51]. Interestingly, some transgenic mice survived, and analysis of the developed regenerating liver nodules revealed that the cells inside the nodules were aneuploid and carried inactivating mutations [51]. This is in line with the assumption that aneuploidy might be beneficial for adaptation processes, which, for instance, has also been demonstrated in fungi [109,110,111,112,113]. Recently, Matsumoto and colleagues suggested that ploidy reduction/HST of polyploid hepatocytes concomitant with subsequent re-polyploidization might play a role in regenerative processes in the liver [51], which could be another explanation for why aneuploidy is tolerated in hepatocytes.

The above summarized findings nicely illustrate the two diametrically opposed sides of cell fusion-induced aneuploidy and genomic instability. However, it must be borne in mind that these data cannot be generalized, which means that not every cell fusion-derived aneuploid and genomically unstable cell would undergo malignant transformation or would be more resistant to stress conditions. In fact, cell fusion-derived aneuploidy and genomic instability are associated with impaired proliferation and overall decreased viability of cells. Several studies demonstrated that aneuploid cells were less proliferative [114,115], more apoptotic [116,117,118], or became senescent [119,120,121], which can most likely be attributed to impaired cellular homeostasis due to altered gene and protein expression levels (for review see [47]). Moreover, even in the context of “cell fusion in cancer”, it has been shown that approximately 99% of tumor cell × normal cell hybrids have died or become senescent. In contrast, only approximately 1% of such hybrids have survived and were able to proliferate [122,123,124]. Interestingly, Wang and colleagues observed that some prostate cancer cell × stromal cell hybrids remained in a quiescent state for up to 8 weeks before starting to proliferate again [124]. This result is still not clear, and future studies should examine what internal processes have caused these cells to start dividing again. Nonetheless, the authors concluded from their data that the principle fate of cancer × stromal hybrids was death [124].

## 5. Conclusions

The biological phenomenon of cell fusion plays a crucial role in various physiological processes, including fertilization, placentation, myogenesis, osteoclastogenesis, and tissue regeneration and wound healing (for review see [34,55,56]). However, even though cell fusion is a widespread biological phenomenon, it is still not fully understood. In accordance with the yet unknown molecules/conditions that direct the merging of two or more cells, it also remains to be elucidated how the process of ploidy reduction/HST is regulated/induced in polyploid hybrid cells and what the fate of cell fusion-derived aneuploid and genomically unstable cells is. As summarized above, ploidy reduction/HST could either give rise to daughter cells with a diploid karyotype [37,42] or to daughter cell that are aneuploid and genomically unstable [7,36,39,45,46,47,48], suggesting that this process might be differentially regulated in distinct cell types. Likewise, aneuploidy and genomic instability appear to be more tolerated in proliferating hepatocytes [36,39,40,49,50,51], whereas in other cell types, aneuploidy and genomic instability are associated with cell death or senescence [117,118,119,120,121]. Again, it remains to be elucidated how these different cellular outcomes (tolerance/viability vs. apoptosis/senescence) are regulated in distinct cell types. Finally, the role of cell fusion in the neoplastic transformation of cells needs to be clarified. A few studies have already shown that neoplastic cells could originate from hybridization events of non-transformed cells [52,53,54], but it remains to be examined whether this might be a common cancer-related mechanism or whether it is restricted to neoplasms that are associated with chronic inflammatory conditions. Increased levels of mutagenic radical oxygen and nitrogen species are present in chronic inflammatory conditions, which also represent a cell fusion friendly milieu, suggesting that such inflammation-related radicals could directly react with newly formed cell fusion-derived hybrids.

In summary, much more work has to be done in the context of cell fusion, including finding a better understanding of how this process is directed, how the process of ploidy reduction/HST is regulated, and what the fate of cell fusion-derived hybrids is.

## Abbreviations

BMSC	bone marrow-derived stem cells
HST	heterokaryon-to-synkaryon transition
MSCs	mesenchymal stem/stromal cells
HSCs	hematopoietic stem cells
GFP	green fluorescent protein
Fah	fumarylacetoacetate hydrolase
NTBC	2-(2-nitro-4-trifluoro-methylbenzoyl)-1,3-cyclohexanedione
IL-4	interleukin-4
RANKL	receptor activator of NF-κB ligand
MMP-9	matrix metallopeptidase 9
DC-STAMP	dendrocyte expressed seven transmembrane protein
OC-STAMP	osteoclast stimulatory transmembrane protein
TNF-α	tumor necrosis factor-α
